# Machine Learning–Based Hyperglycemia Prediction: Enhancing Risk Assessment in a Cohort of Undiagnosed Individuals

**DOI:** 10.2196/56993

**Published:** 2024-09-11

**Authors:** Kolapo Oyebola, Funmilayo Ligali, Afolabi Owoloye, Blessing Erinwusi, Yetunde Alo, Adesola Z Musa, Oluwagbemiga Aina, Babatunde Salako

**Affiliations:** 1 Nigerian Institute of Medical Research, Lagos, Nigeria; 2 Centre for Genomic Research in Biomedicine, Mountain Top University, Ibafo, Nigeria

**Keywords:** hyperglycemia, diabetes, machine learning, hypertension, random forest

## Abstract

**Background:**

Noncommunicable diseases continue to pose a substantial health challenge globally, with hyperglycemia serving as a prominent indicator of diabetes.

**Objective:**

This study employed machine learning algorithms to predict hyperglycemia in a cohort of individuals who were asymptomatic and unraveled crucial predictors contributing to early risk identification.

**Methods:**

This dataset included an extensive array of clinical and demographic data obtained from 195 adults who were asymptomatic and residing in a suburban community in Nigeria. The study conducted a thorough comparison of multiple machine learning algorithms to ascertain the most effective model for predicting hyperglycemia. Moreover, we explored feature importance to pinpoint correlates of high blood glucose levels within the cohort.

**Results:**

Elevated blood pressure and prehypertension were recorded in 8 (4.1%) and 18 (9.2%) of the 195 participants, respectively. A total of 41 (21%) participants presented with hypertension, of which 34 (83%) were female. However, sex adjustment showed that 34 of 118 (28.8%) female participants and 7 of 77 (9%) male participants had hypertension. Age-based analysis revealed an inverse relationship between normotension and age (*r*=−0.88; *P*=.02). Conversely, hypertension increased with age (*r*=0.53; *P*=.27), peaking between 50‐59 years. Of the 195 participants, isolated systolic hypertension and isolated diastolic hypertension were recorded in 16 (8.2%) and 15 (7.7%) participants, respectively, with female participants recording a higher prevalence of isolated systolic hypertension (11/16, 69%) and male participants reporting a higher prevalence of isolated diastolic hypertension (11/15, 73%). Following class rebalancing, the random forest classifier gave the best performance (accuracy score 0.89; receiver operating characteristic–area under the curve score 0.89; *F*_1_-score 0.89) of the 26 model classifiers. The feature selection model identified uric acid and age as important variables associated with hyperglycemia.

**Conclusions:**

The random forest classifier identified significant clinical correlates associated with hyperglycemia, offering valuable insights for the early detection of diabetes and informing the design and deployment of therapeutic interventions. However, to achieve a more comprehensive understanding of each feature’s contribution to blood glucose levels, modeling additional relevant clinical features in larger datasets could be beneficial.

## Introduction

Noncommunicable diseases (NCDs) have become a substantial public health concern in Africa [1]. Conditions like coronary artery disease, stroke, hypertension, and diabetes, which were once primarily associated with high-income nations or affluence, have now become pervasive health challenges in low- and middle-income countries and across diverse socioeconomic strata [1]. The complex nature of NCDs underscores the need for a comprehensive approach to risk assessment, intervention, and prevention.

Suburban communities serve as a distinctive microcosm within an evolving landscape of diseases [2,3]. These communities, characterized by the coexistence of traditional and modern lifestyles, grapple with risk factors that necessitate thorough examination [4]. The epidemiological shift from communicable to NCDs, coupled with limited health care resources, especially in suburban parts of low- and middle-income countries [5,6], stresses the importance of this research. In addition, recent advancements in genetic research have elucidated the underlying mechanisms of various complex NCDs. The identification of individuals at an elevated genetic risk for NCDs has the potential to revolutionize the approach of health care stakeholders to disease management. However, the effective implementation of genetic screening for NCD risk analysis relies on a robust understanding of the baseline contributors prevalent in the target population [7,8]. This study provided a comprehensive description of the prevalence and intricate interplay of risk factors associated with NCDs, highlighting hypertension, obesity, and diabetes. The specific focus was on undiagnosed individuals who were asymptomatic to elucidate the complex relationships of these health indicators within this population.

Machine learning encompasses a diverse set of algorithms designed to extract patterns from data and establish associations between these patterns and discrete sample classes within the data. Machine learning proves to be a valuable tool for identifying potential disease risk factors, elucidating etiology, and interpreting complex pathological processes in the context of NCDs [9-16]. In this study, multiple machine learning algorithms were developed to predict elevated blood glucose levels in a cohort of undiagnosed individuals who were asymptomatic. The primary objective was to systematically compare the accuracies of supervised machine learning classifiers to identify the most effective model for predicting hyperglycemia. Leveraging the predictors in the dataset, we meticulously constructed and evaluated these models for the identification of significant features associated with potential diabetes in the population.

## Methods

### Ethical Considerations

Ethical approval was obtained from the institutional review board of the Nigerian Institute of Medical Research (IRB/21/074). Data collected from participants was anonymized, and personal identifiers were removed. Furthermore, participants’ data were stored in our database with access restricted to authorized research personnel only. The study participants received refreshments as compensation for their time and contribution. This gesture was intended to acknowledge their involvement and ensure their comfort during the study sessions while maintaining fairness and transparency in the compensation process.

### Participant Recruitment and Screening

This study was carried out as part of a parallel community-based genetic screening of apparently healthy adults living in Ijede Community, Lagos, Nigeria. Following informed consent, participants were recruited, and 10 ml of venous blood samples were collected per participant. Demographic information, BMI, knowledge, attitude, and practices were obtained from the participants. The study clinician also obtained the participants’ personal and family medical history as well as their smoking status. Exclusion criteria included pregnancy at the time of recruitment, placement on antihypertensive or antidiabetic chemotherapy or radiotherapy, current or previous hematologic or tumoral diseases, and known chronic diseases. Participants underwent electrocardiogram (ECG) screening (SonoHealth, United States) to provide clues on heart defects or other heart-related problems. Hemoglobin electrophoresis was conducted to detect possible hemoglobinopathy in the participants [17]. In addition, random blood glucose (RBG) concentrations (Guilin Royalze, China) and blood pressure (BP) values (Iston Mediq, United States) were determined to evaluate the presence or absence of prediabetes, diabetes, prehypertension, or hypertension onset in the participants. Participants with screening tests outside normal ranges were advised to visit their health care specialists for further checks. Normal BP was described as systolic BP (SBP) <120 mmHg and diastolic BP (DBP) <80 mmHg. Elevated BP was defined as SBP 120‐129 mmHg and DBP <80 mmHg, stage 1 hypertension (prehypertension) as SBP ≥130‐139 mmHg and DBP 80‐89 mmHg, and stage 2 hypertension as SBP ≥140 and DBP ≥90 mmHg [18]. Isolated systolic hypertension (ISH) was described as SBP >140 mmHg and DBP <90 mmHg [19]. Isolated diastolic hypertension (IDH) is an important subtype of hypertension defined as SBP <130 mmHg and DBP ≥80 mmHg [20]. Prediabetes was defined as an RBG concentration of 140‐199 mg/dl or fasting blood glucose of 100‐125 mg/dl. Diabetes mellitus was defined as an RBG level ≥200 mg/dl or fasting blood glucose level ≥126 mg/dl [21]. However, as all the participants reported that they were not fasting, RBG values were documented.

### Correlation Analysis

Data cleaning, exploratory analysis, and feature engineering were performed in Google Colab (with Python 3.10; Python Software Foundation). The target variable was specified as “blood glucose,” where 1 indicated an RBG concentration ≥140 mg/dl and 0 indicated an RBG concentration <140 mg/dl. Independent variables included age (integer), sex (integer), BMI (float), smoking status (integer), ECG (float), hemoglobin (float), cholesterol (float), uric acid (float), SBP (integer), DBP (integer), normal BP (integer), elevated BP (integer), prehypertension (integer), hypertension (integer), ISH (integer), IDH (integer), prediabetes (integer), diabetes (integer), normal glucose (integer), abnormal ECG values (integer), and normal ECG values (integer). The dataset was checked and visualized for missingness using seaborn heatmap (Figure S1 in Multimedia Appendix 1). Missing values were replaced with column mean (for continuous variables) or mode (for categorical variables). Duplicate rows and outliers were dropped before encoding categorical variables and creating dummy variables. Subsequently, we created a heatmap for the correlation of independent variables with the target column in descending order. The cleaned dataset was then scaled for subsequent training of machine learning models. A *P* value ≤.05 was considered statistically significant.

### Machine Learning Algorithms and Evaluation

**Figure 1.**
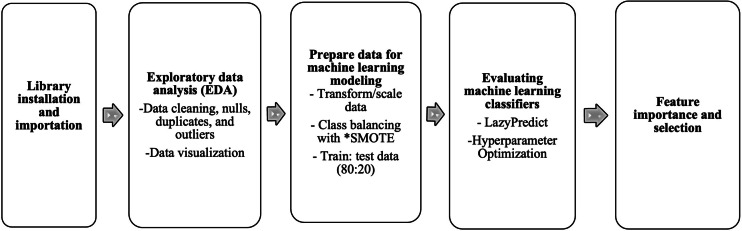
Pipeline for model development. SMOTE: synthetic minority oversampling technique.

The study adopted 26 supervised classification algorithms and compared their accuracies to identify the best-performing model for predicting high blood glucose, which was defined in this study as an RBG concentration ≥140 mg/dl (Figure 1). Specifically, after the installation and importation of Sci-Kit Learn libraries [22], we carried out data cleaning, exploration, and scaling to improve the efficiency of our model (Multimedia Appendix 1). Imbalances in the distribution of hyperglycemia cases and noncases within the dataset might affect the model’s performance. Addressing this imbalance and validating the model on balanced datasets could enhance its robustness. To address the class imbalance in the outcome variable (blood glucose level), we adopted the synthetic minority oversampling technique (SMOTE). SMOTE tackled the underrepresentation of the minority class and rebalanced the class distribution for equitability [23]. After resampling, we split the data into training and test sets at a ratio of 80:20, respectively, using the train_test_split function in Sci-Kit Learn. We went further to select and rank the performances of the machine learning algorithms using LazyPredict to obtain the weighted average of the *F*_1_-scores and accuracy scores as well as the receiver operating characteristic–area under the curve (ROC-AUC) score. For hyperparameter optimization, we adopted GridSearchCV [24]. The grid search technique constructs many versions of the model with all possible combinations of hyperparameters to return the best one [25]. Subsequently, we determined feature importance to provide insight into which features are most associated with elevated blood glucose levels using the best-performing model. To operationalize the best-performing model generated at scale, the training file was stored as a serialized pickle file. Subsequently, we used the Fast Application Programming Interface in Google Colab [26] to make an inference call from the model using the predict() function and generated our application programming interface. Pyngrok was used to open secure tunnels from public URLs to the local host.

## Results

### Cohort Description

**Figure 2.**
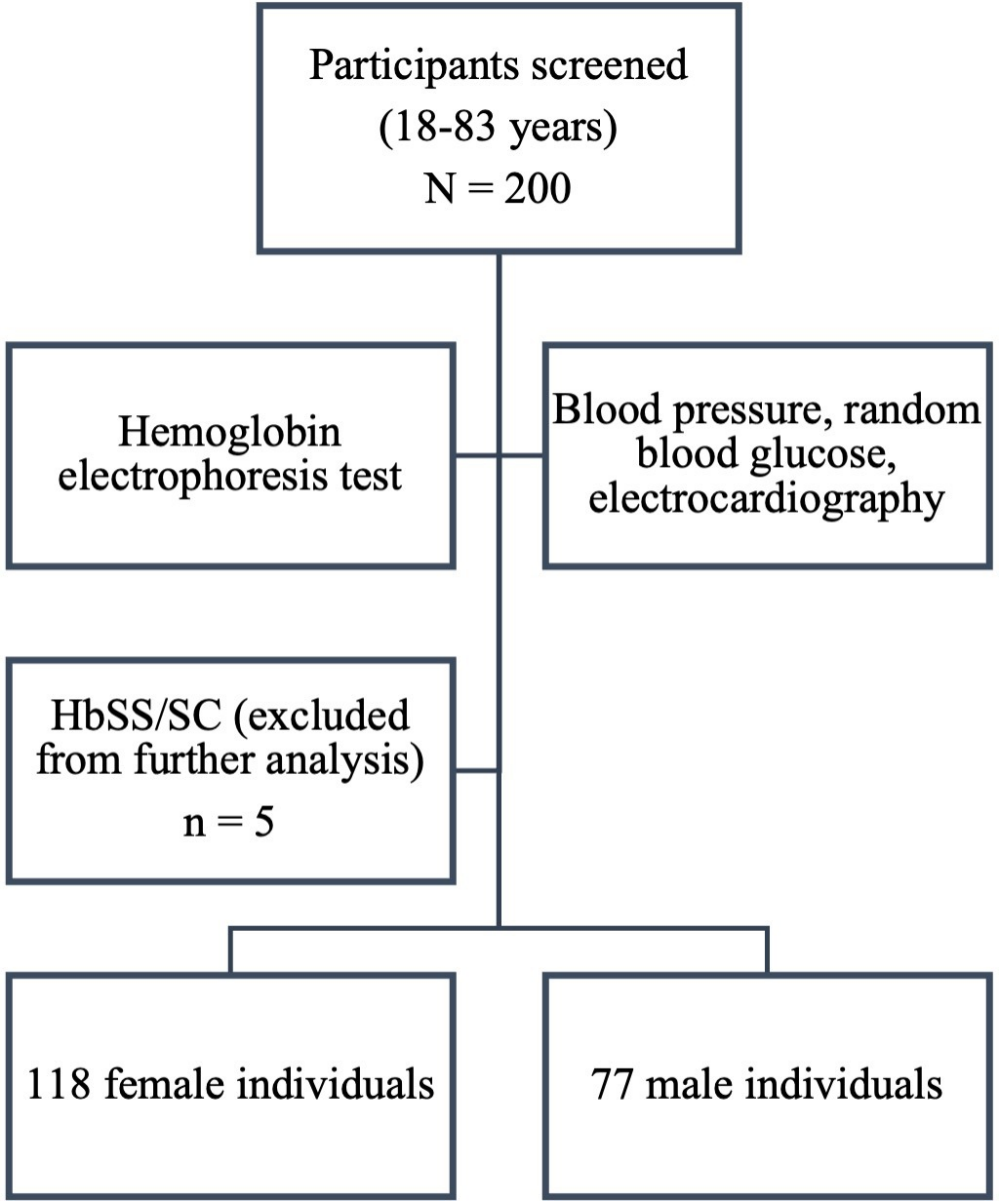
Participant recruitment and screening.

A total of 200 participants ages 18‐83 years were enrolled in the cohort. However, after hemoglobin electrophoresis screening, 5 participants were found to possess the hemoglobins SS and SC genotypes and were excluded from further analysis. A total of 118 female and 77 male participants were included (Figure 2 and Figure S2 in Multimedia Appendix 1).

### Correlation Analysis

**Figure 3.**
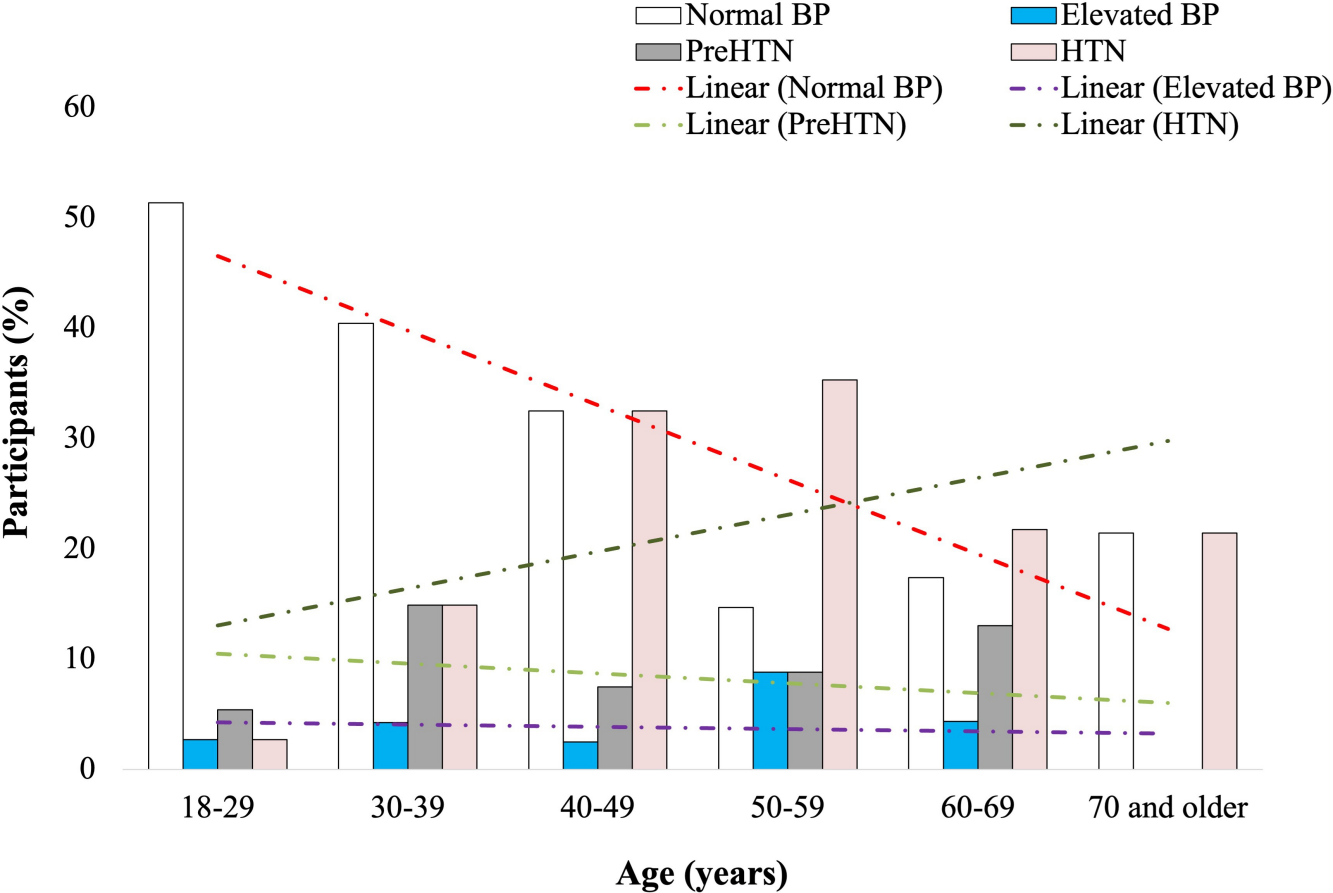
Age-based analysis of BP. Percentage of participants with normal BP reduced with increases in age (*r*=−0.88; *P*=.02). Prevalence of HTN increased with age (*r*=0.53; *P*=.27), peaking between 50-59 years. BP: blood pressure; HTN: hypertension.

**Figure 4.**
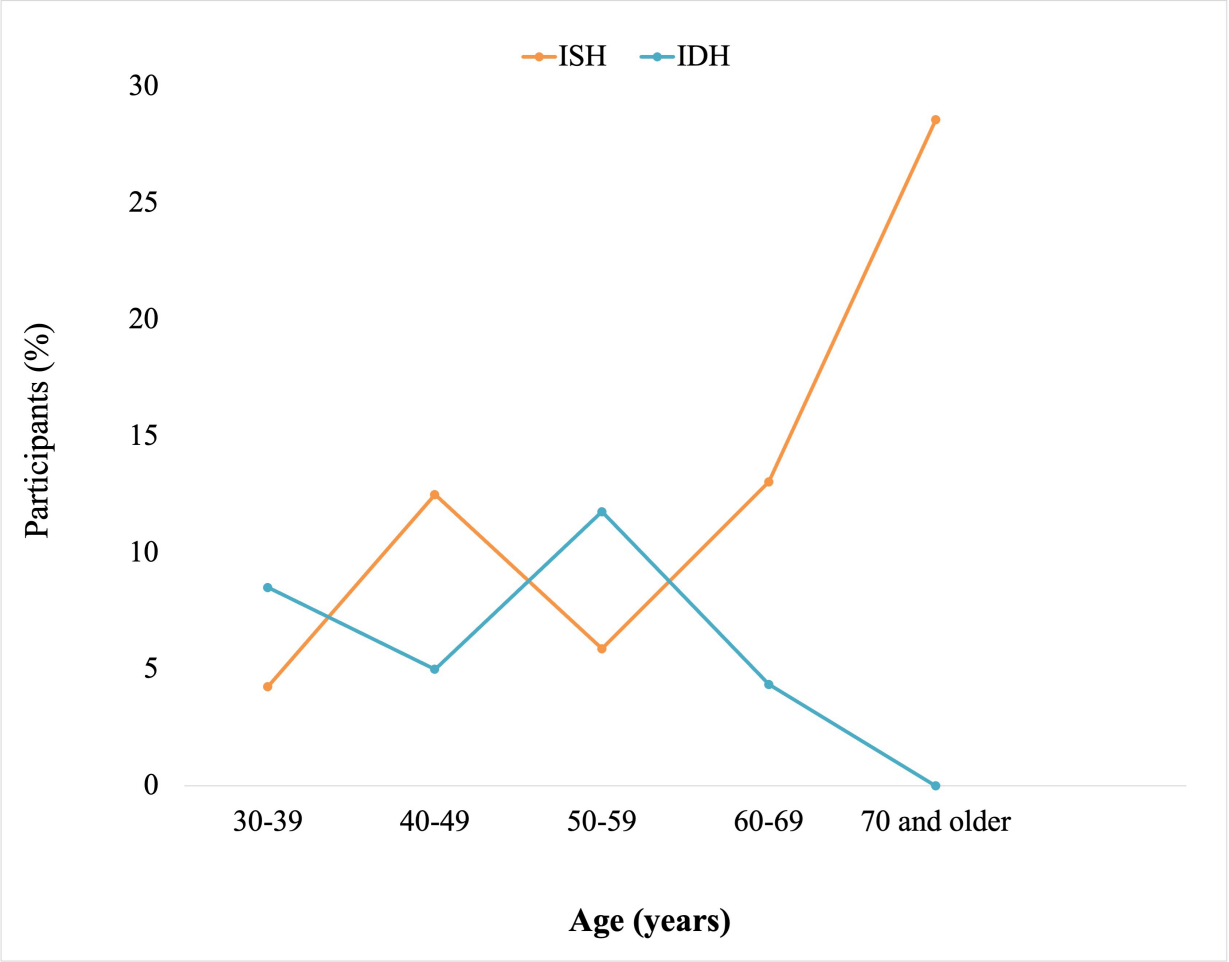
Age-based analysis of ISH and IDH. ISH increased with participants’ age (*r*=0.86; *P*=.03), unlike IDH (*r*=−0.71; *P*=.11). IDH: isolated diastolic hypertension; ISH: isolated systolic hypertension.

**Figure 5.**
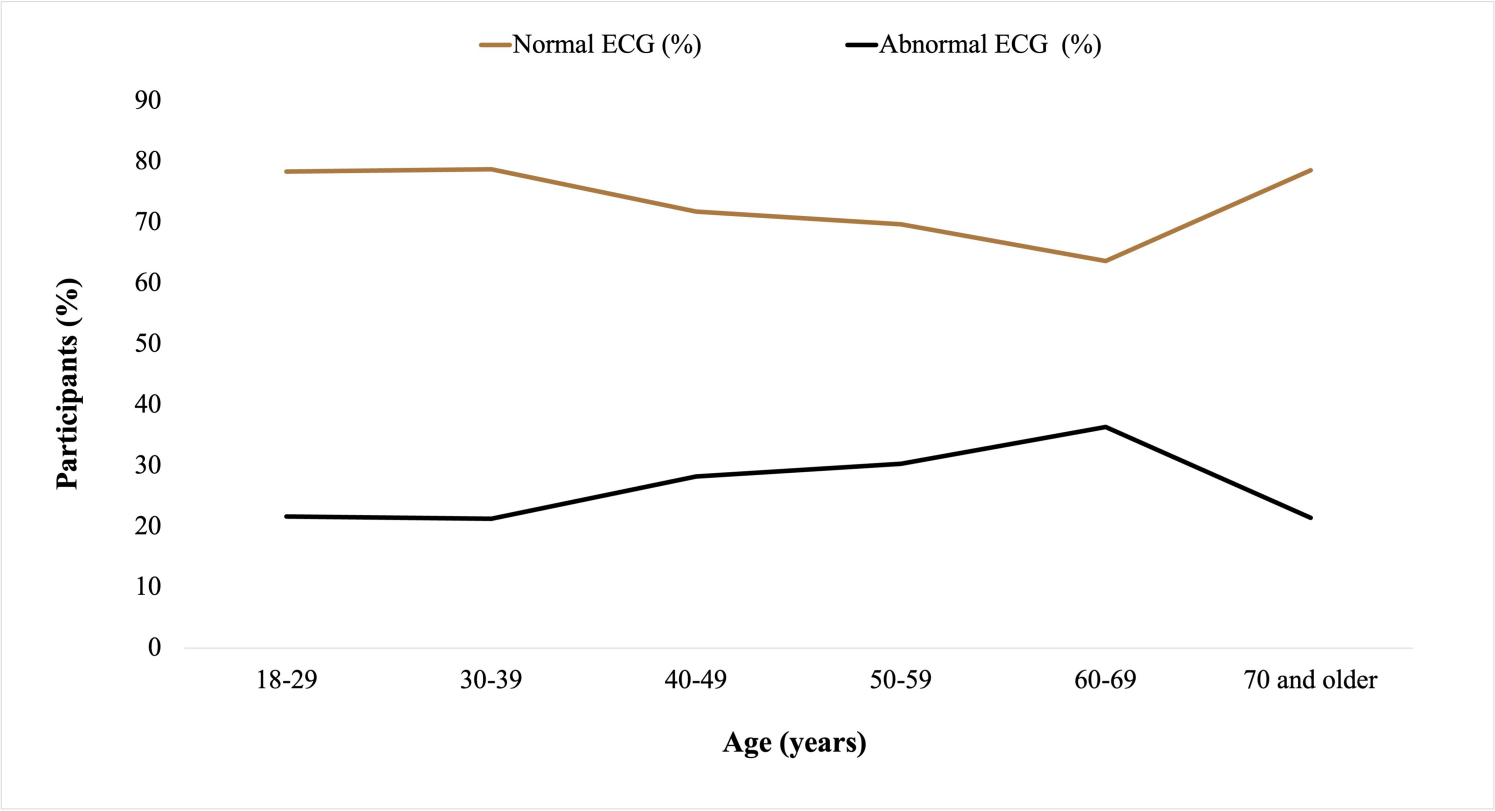
Age-based ECG analysis. Age-dependent increase in the percentage of participants with abnormal ECG values peaking between ages 60-69 years. ECG: electrocardiogram.

**Figure 6.**
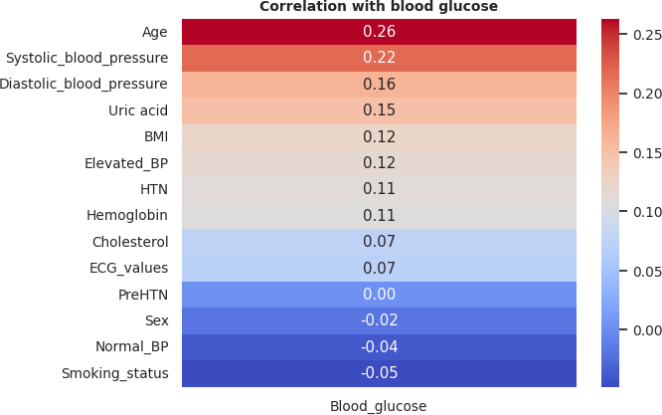
Correlation matrix of independent variables with the outcome variable. BP: blood pressure; ECG: electrocardiogram; HTN: hypertension.

Participants were categorized into six age groups: 18‐29, 30‐39, 30‐49, 50‐59, 60‐69, and ≥70 years. Of the 195 participants, normal BP, elevated BP, and prehypertension were recorded in 63 (32.3%), 8 (4.1%), and 18 (9.2%) participants, respectively. A total of 41 (21%) participants presented with hypertension, of which 34 (83%) were female (Figure S3 in Multimedia Appendix 1). Age-based analysis revealed an inverse relationship between normotension and age (*r*=−0.88; *P*=.02). Consistently, hypertension increased with age (*r*=0.53; *P*=.27), peaking between 50‐59 years (Figure 3). Of the 195 participants, ISH and IDH were recorded in 16 (8.2%) and 15 (7.7%) participants, respectively, with female participants recording a higher prevalence of ISH (11/16, 69%) and male participants reporting a higher prevalence of IDH (11/15, 73%; Figure S4 in Multimedia Appendix 1). There was a positive correlation between ISH and participants’ age (*r*=0.86; *P*=.03), whereas IDH was inversely correlated with age (*r*=−0.71; *P*=.11; Figure 4). We went further to examine the heart rates of the participants and observed an age-dependent increase in the percentage of participants with abnormal ECG values peaking between ages 60‐69 years (Figure 5). However, no significant difference was observed in the ECG values of male and female participants ($\sqrt{X^{2}}$=0.13; *P*=.72; Figure S5 in Multimedia Appendix 1). An RBG value between 140‐199 mg/dl (prediabetes) was detected in 22 (11.3%) and diabetes was suspected in 5 (2.6%) of the 195 participants. A total of 163 (85.8%) participants had normal blood glucose. Though not statistically significant, an inverse relationship (*r*=−0.81; *P*=.06) was observed between age and normal glucose level, and the frequency of prediabetes (*r*=0.63; *P*=.19) and suspected diabetes (*r*=0.58; *P*=.24) seemed to increase with age (Figure S6 in Multimedia Appendix 1). Meanwhile, a correlation matrix between each independent variable and the target column (blood glucose level) showed that age had the highest ranking even though the correlation coefficient was weak (Figure 6 and Figure S7 in Multimedia Appendix 1).

### Machine Learning Algorithms and Evaluation

**Figure 7.**
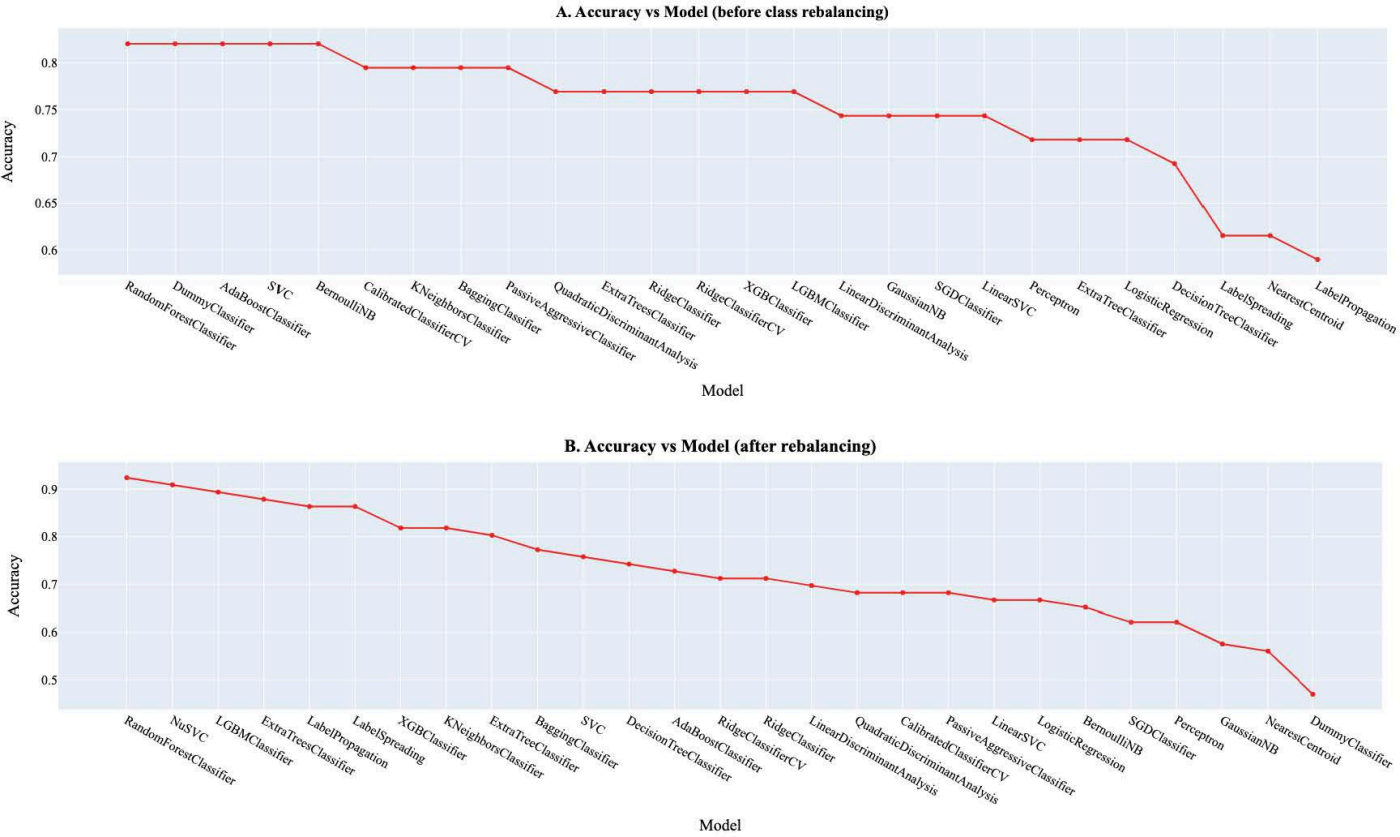
Accuracy scores of machine learning classifiers (A) before class rebalancing with the synthetic minority oversampling technique and (B) after class rebalancing with the synthetic minority oversampling technique. CV: cross-validation; LGBM: light gradient boosting machine; NB: naive Bayes; SGD: stochastic gradient descent; SVC: support vector classification; XGB: extreme gradient boosting.

Following data cleaning, transformation (Figure S8 in Multimedia Appendix 1), and observation of a class imbalance in the target variable (Figure S9 in Multimedia Appendix 1), whereby the raw dataset demonstrated that 163 (83.6%) of the 195 participants had normal blood glucose (0), while 32 (16.4%) had a high blood glucose level (1), rebalancing was established with SMOTE to yield an even representation of both categories of blood glucose level (counter: 0: 163; 1: 163). When the performance of each classifier was tested, the reports showed that the random forest classifier (Figures7  8) gave the best accuracy (accuracy score 0.89; ROC-AUC score 0.89; *F*_1_-score 0.89), followed by extra trees (accuracy score 0.88; ROC-AUC score 0.88; *F*_1_-score=0.88) and extreme gradient boosting classifiers (accuracy score 0.86; ROC-AUC score 0.86; *F*_1_-score 0.86; Figure 7B and Table S2 in Multimedia Appendix 1).

To determine the importance of each variable (feature) to the outcome (blood glucose level), we carried out a random forest feature analysis. The importance of a feature is calculated based on how much the tree nodes that use that feature reduce impurity across all trees in the forest. The key findings showed that uric acid and age were the most important features associated with elevated blood glucose (Table 1), followed by SBP and BMI.

**Figure 8.**
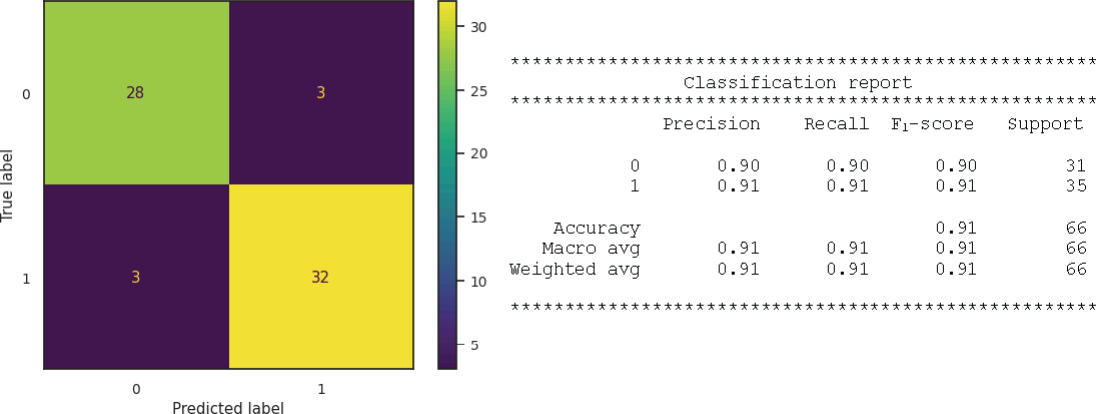
Random forest confusion matrix showing a visual representation of the true vs predicted labels. True positive: the values that were positive and were predicted positive, that is, 31 cases of hyperglycemia were predicted correctly by the model. False positive: the values that were negative but falsely predicted as positive. In this case, only 3 cases were false positives. False negative: the values that were positive but falsely predicted as negative. In this instance, there were 4 false negatives. True negative: the values that were negative and were predicted negative. Here, 28 cases were detected. In all, the weighted average of the accuracy score and *F*_1_-score were 0.89 and 0.89, respectively. Precision is a metric that quantifies the accuracy of a classifier by determining the number of correctly identified members of a class divided by all instances where the model predicted that specific class. In the context of hyperglycemia prediction, precision would be the count of accurate predictions of hyperglycemia divided by the total instances where the classifier predicted “hyperglycemia,” regardless of correctness. Recall, on the other hand, measures the effectiveness of a classifier in correctly identifying members of a class by dividing the number of correctly identified instances by the total number of actual members in that class. In the hyperglycemia scenario, recall would represent the number of actual participants with hyperglycemia correctly identified by the classifier. The *F*_1_-score is a composite metric that combines both precision and recall into a single value. It provides a concise evaluation of a classifier’s performance. A high *F*_1_-score indicates that both precision and recall are high, while a low *F*_1_-score suggests that one or both metrics are low. This metric is particularly useful for quickly assessing whether a classifier effectively identifies members of a class or if it resorts to shortcuts, such as indiscriminately classifying everything as a member of a larger class. avg: average.

**Table 1.** Blood glucose predictors.

Feature	Importance^a^
Age	0.17
Uric acid	0.17
BMI	0.12
Systolic blood pressure	0.11
Diastolic blood pressure	0.10
Cholesterol	0.09
Hemoglobin	0.08
Electrocardiogram values	0.04
Sex	0.04
Normal blood pressure	0.03
Elevated blood pressure	0.02
Hypertension	0.02
Pre-hypertension	0.01
Smoking status	0.01

a The importance of a feature is calculated based on how much the tree nodes that use that feature reduce impurity across all trees in the forest.

## Discussion

### Principal Findings

NCDs, such as cancer, cardiovascular diseases, and diabetes, are progressively becoming the primary causes of mortality in sub-Saharan Africa [27]. This epidemiological shift is primarily attributed to limitations in implementing crucial control measures, such as prevention and early detection [1]. This research focused on exploring key clinical indices of NCDs in individuals who are asymptomatic. The application of machine learning in disease prediction is now well established for its immense potential in analyzing complex datasets and uncovering patterns that may elude human detection [28-31]. This investigation employed various machine learning algorithms to predict hyperglycemia to enable early identification of individuals at risk of developing diabetes. The study identified suspected hypertension in 21% of study participants, underscoring the urgency of addressing hypertension as a major health challenge in the country. Furthermore, a notable increase in the prevalence of hypertension with advancing age was observed. However, the investigation into hypertension subtypes revealed a dual phenomenon: a pronounced increase in systolic hypertension with age and a concomitant reduction in diastolic hypertension.

Several factors may contribute to the observed age-related increase in systolic hypertension. Physiological changes, alterations in vascular reactivity, and lifestyle factors could play decisive roles in driving the upward trajectory of SBP with advancing age [32,33]. In contrast, the age-related reduction in diastolic hypertension may be associated with changes in arterial compliance, heart rate dynamics, or other physiological adaptations over the aging process [34]. Recognizing these dual dynamics holds significant clinical implications, necessitating tailored screening protocols and interventions to address the unique challenges posed by hypertension in different age groups.

Moreover, a sex disparity was observed, with systolic hypertension being more prevalent in female participants and diastolic hypertension being more common in male participants. This sex difference may be linked to heart rate variability or hormonal influences, particularly fluctuations in estrogen levels in female individuals. However, understanding how blood vessels respond to changes in pressure and the potential impact on SBP would be crucial in deciphering these sex disparities [35-37]. Therefore, tailoring screening protocols and interventions to address the unique challenges posed by hypertension in different age groups and sexes is essential to mitigate the overall burden of this condition.

ECG is a pivotal tool for assessing cardiac health, and its interpretation can provide valuable insights into cardiovascular conditions. Our investigation revealed a remarkable age-dependent pattern in abnormal ECG values, reaching a peak at 70 years. Advancing age often coincides with a myriad of physiological changes, including alterations in cardiac structure and function [38-40]. A comprehensive exploration of these factors is essential for delineating the intricate relationship between aging and abnormal ECG findings.

The global burden of diabetes is well-documented [41-43], but our investigation into supposedly healthy individuals has unearthed a concerning revelation. Despite outward appearances of health, there existed a relatively high prevalence of suspected prediabetes and diabetes in the cohort. This underscores the importance of probing beyond outward health markers to understand the latent metabolic landscape [44-47]. This prompts a reevaluation of health screening protocols to incorporate metabolic parameters in apparently healthy populations. Early detection and intervention strategies should be tailored to encompass metabolic assessments, providing an opportunity for targeted preventive measures and lifestyle modifications.

In the realm of predictive modeling, selecting the most effective machine learning algorithm is paramount. Our study, aimed at evaluating various algorithms, revealed insightful findings regarding their predictive performances. Upon meticulous evaluation, random forest emerged as the top-performing algorithm, consistently delivering the highest accuracy among the tested models. The success of the random forest algorithm can be attributed to its ensemble learning nature [48,49], which harnesses the collective power of multiple decision trees. This enables robustness against overfitting, enhanced generalization, and effective handling of complex datasets with diverse features. The observed superiority of random forest in our study has profound implications for future applications, suggesting its applicability across diverse datasets and underscoring its potential as a reliable choice for achieving high predictive accuracy.

To investigate the intricate determinants of hyperglycemia, our study employed a robust feature importance analysis, with compelling results showcasing uric acid and age as the most influential predictors. Uric acid’s prominence as a predictor of hyperglycemia adds a unique dimension to our understanding of metabolic health. While traditionally associated with conditions like gout, our findings suggest a potential link between hyperuricemia and hyperglycemia, urging further exploration into the underlying physiological mechanisms. The identification of age as a key predictor aligns with existing knowledge regarding the age-associated risk of hyperglycemia [49-51]. Our findings reinforce the significance of age as a robust indicator, reflecting the cumulative impact of aging processes on metabolic health and glucose regulation. The recognition of uric acid and age as pivotal predictors holds significant clinical implications. Health care practitioners can leverage these findings to enhance risk assessment strategies for hyperglycemia. Incorporating uric acid measurements and age considerations into routine screenings may facilitate early identification of individuals at heightened risk, enabling proactive interventions. While our study sheds light on the importance of uric acid and age, further research is warranted to unravel the intricate relationships and mechanisms underlying these associations. Longitudinal studies exploring the dynamic interplay between uric acid, age, and hyperglycemia can deepen our understanding and inform targeted interventions.

### Limitations and Future Direction

While our study provides valuable insights into predicting hyperglycemia using machine learning in undiagnosed individuals, it is essential to acknowledge certain limitations that may impact interpretation. First, the size of our cohort may limit the generalizability of the results. A larger and more diverse sample could enhance the external validity of the predictive model. Furthermore, the study did not account for potential variations in clinical practice, including differences in diagnostic criteria. For instance, the study did not take into consideration orthostatic hypotension, a decrease in SBP ≥20 mmHg or a DBP decrease of ≥10 mmHg within 3 minutes of standing, especially in older individuals [19]. Although seats were provided to participants, we could not accurately document how long participants had been standing before attending the screening. Besides, phenomena such as postprandial hypotension (a reduction in BP after meals, a common cause of syncope and falls in older individuals who are healthy and have hypertension), circadian BP variability, and white-coat (nonsustained) hypertension, especially in older adults, were not factored into the analyses [52-54]. As such, incorporating standardized criteria across diverse health care settings could enhance our model’s clinical applicability.

Moreover, the study did not dissect the influence of ethnicity and genetics on hyperglycemia [55,56]. Future research could explore these aspects to provide a more comprehensive understanding of predictive factors. Since the dataset primarily comprises information from a specific geographic location or demographic group, extrapolating the findings to other populations requires caution as regional variations in lifestyle, genetics, and health care practices may influence the performance of the predictive model. In addition, the cross-sectional nature of our study limits our ability to establish causation or assess changes over time. Therefore, longitudinal studies would be beneficial to understand the dynamic nature of hyperglycemia predictors. The model’s performance was evaluated on the same dataset used for training, raising the potential for overfitting. External validation on an independent dataset would be crucial to assess its generalizability and reliability in real-world scenarios. Lastly, the importance of a feature in a random forest model does not necessarily mean a causal relationship and other models might find different results if additional features are introduced. Future approaches are expected to accommodate more features and larger datasets. This will account for the deployment of built and containerized models as publicly accessible web applications. Nevertheless, this study has expounded the potential of machine learning for early disease detection, risk assessment strategies, proactive interventions, and targeted therapeutic design.

### Conclusions

This study has made a substantial contribution to the expanding domain of predictive modeling and offers promising implications for enhancing early detection and personalized risk assessment, particularly in the context of hyperglycemia and its potential association with diabetes. The research has not only brought to light the prevalence of undiagnosed hypertension and isolated systolic and diastolic hypertension but also highlighted factors associated with elevated blood glucose within the population. The findings of this study emphasize the significance of regular screening, effective intervention strategies, and targeted therapeutic designs. Collectively, the results contribute to the overarching effort to enhance health care outcomes through proactive and tailored approaches.

## Supplementary material

10.2196/56993 Multimedia Appendix 1 Supplementary methods and figures.

10.2196/56993 Multimedia Appendix 2 Full dataset.

## Abbreviations

BP: blood pressure
DBP: diastolic blood pressure
ECG: electrocardiogram
IDH: isolated diastolic hypertension
ISH: isolated systolic hypertension
NCD: noncommunicable disease
RBG: random blood glucose
ROC-AUC: receiver operating characteristic–area under the curve
SBP: systolic blood pressure
SMOTE: synthetic minority oversampling technique
