# Supplementary material for: Machine Learning–Based Hyperglycemia Prediction: Enhancing Risk Assessment in a Cohort of Undiagnosed Individuals
Source: JMIRx Med. 2024 Sep 11;5:e56993. doi: 10.2196/56993 (PMC11441453; doi:10.2196/56993)
Supplement: Multimedia Appendix 1 [file xmed-v5-e56993-s001.docx]

Machine Learning-Based Hyperglycemia Prediction: Enhancing Risk Assessment in a Cohort of Undiagnosed Individuals

Kolapo Oyebola^1,2,3^, Funmilayo Ligali^1,2,3^, Afolabi Owoloye^1,2,3^, Blessing Erinwusi^1^, Yetunde Alo^1^, Adesola Musa^2^, Oluwagbemiga Aina^2^ and Babatunde Salako^2^

^1^Centre for Genomic Research in Biomedicine, Mountain Top University, Ibafo, Nigeria

^2^Nigerian Institute of Medical Research, Lagos, Nigeria

**Supplementary Methods**

**Train-test dataset splitting**

This code was used to split the data into training and test sets for a machine learning model (<https://github.com/oyebolakolapo/Machine-Learning-Prediction-of-Elevated-Blood-Glucose-in-a-Cohort-of-Apparently-Healthy-Adults>). First, the code separated the features (x) and target (y) variables from the original dataset bank_data using the drop() method. The drop() method was used to remove a specified column from the dataset. In this case, the column 'y' was removed from the dataset and assigned to the variable x. The column 'y' was assigned to the variable y. Next, the train_test_split() function from the scikit-learn library was used to split the data into training and test sets. The function takes four arguments: the scaled features (X), the target (y), the test size (0.2 in this case), and a random state (which is not specified in this code). The test size argument specifies the proportion of the data that should be used for testing the model. In this case, 20% of the data was used for testing and 80% was used for training. The function returned four variables: X_train, X_test, y_train, and y_test. These variables contained the training and test sets for the features and target variables. The training sets were used to train the machine learning model, and the test sets were used to evaluate the performance of the model.

**Data cleaning steps and outcomes**

The original dataset contained 22 columns and 195 rows, eight continuous variable and 14 categorical variables (Multimedia File 2). The dataset was checked for null values with seaborn heatmap used as a visual scan. For continuous variables, mean values were imputed into missing cells while modes were adopted for categorical variables. Subsequently, “Normal_glucose” and Normal_ECG_Values” were recoded and ‘High” and ‘Normal” values were replaced with “0” and “1”. Columns “Normal_glucose” was then renamed as “Blood_glucose”, while “Normal_ECG_Values” was renamed as “ECG_values”. No duplicate rows were observed. We checked and found no outliers. Next, we had a cursory view of the target variable (Blood_glucose) to check the distribution of the two outcomes “high” and “normal” recoded as “1” and “0” respectively. Count of “0” was 163/195 (83.6%) and 32/195 (16%) for “1”. Data is not balanced hence we applied SMOTE technique much later before deploying machine learning models. Meanwhile, we interrogated how the features compared in a correlation matrix.

**Supplementary Figures**

**
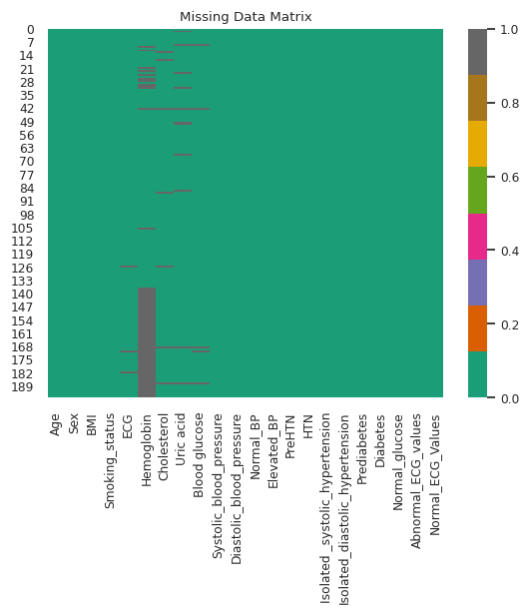

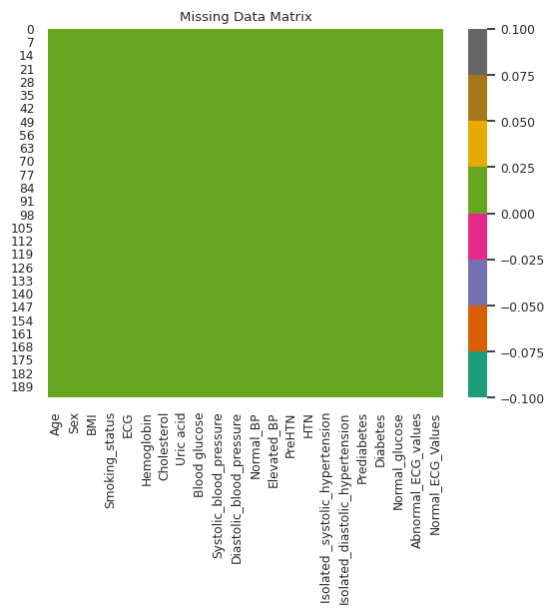
**

B

A

**Fig. S1: Dataset overview before (A) and after (B) missing values were treated.**

**
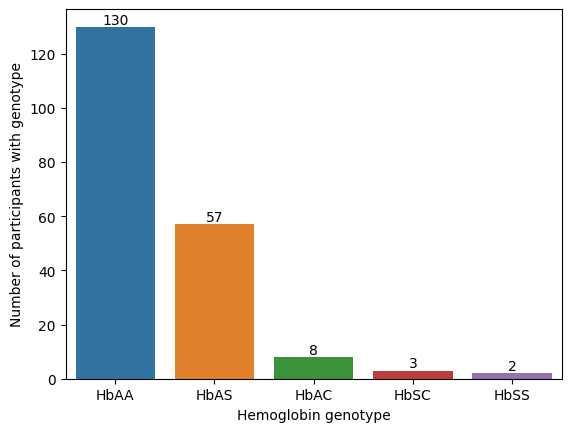
**

**Figure S2: Hemoglobin variants in the cohort**


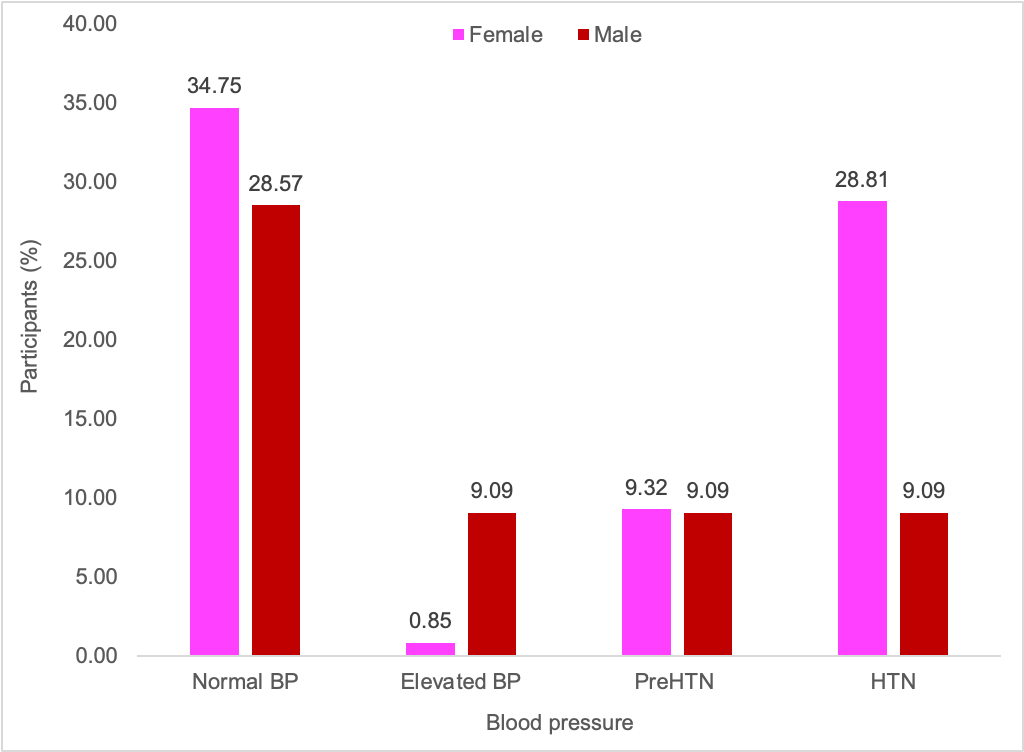


**Figure S3: Blood pressure values recorded in the cohort.**


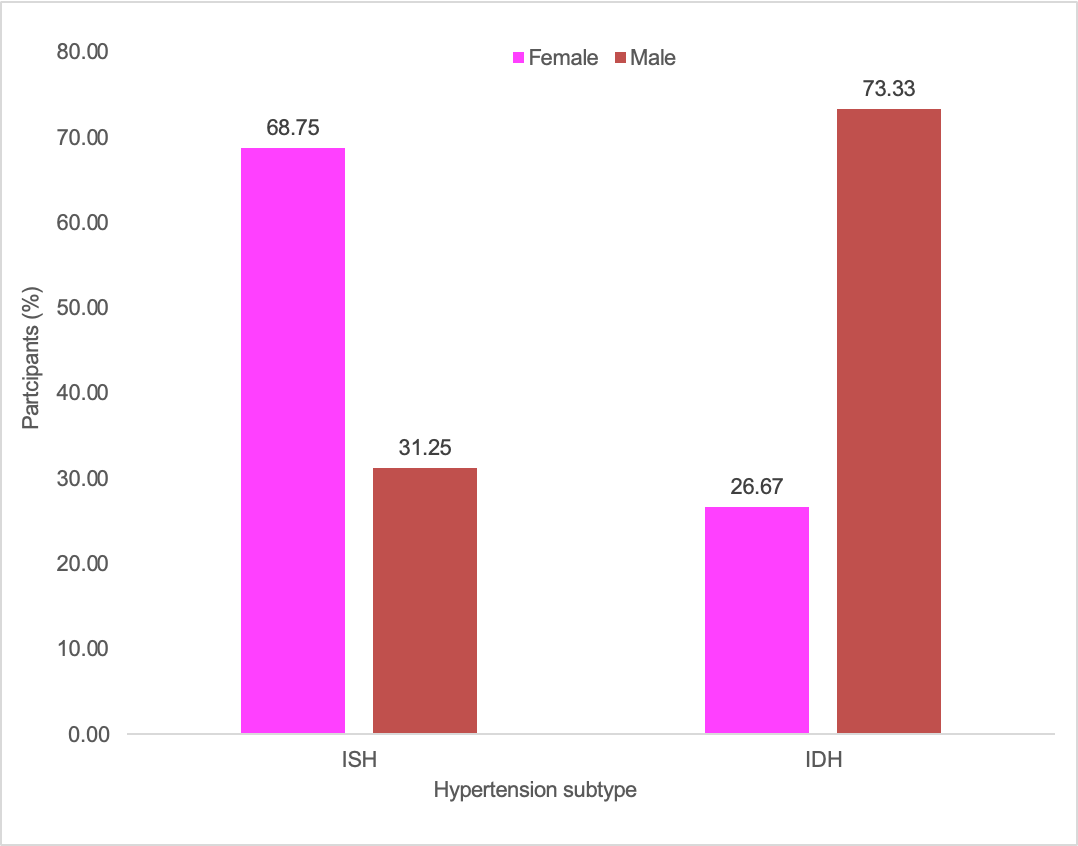


**Figure S4: Prevalence of isolated systolic hypertension (ISH) and isolated diastolic hypertension (IDH) in the cohort**


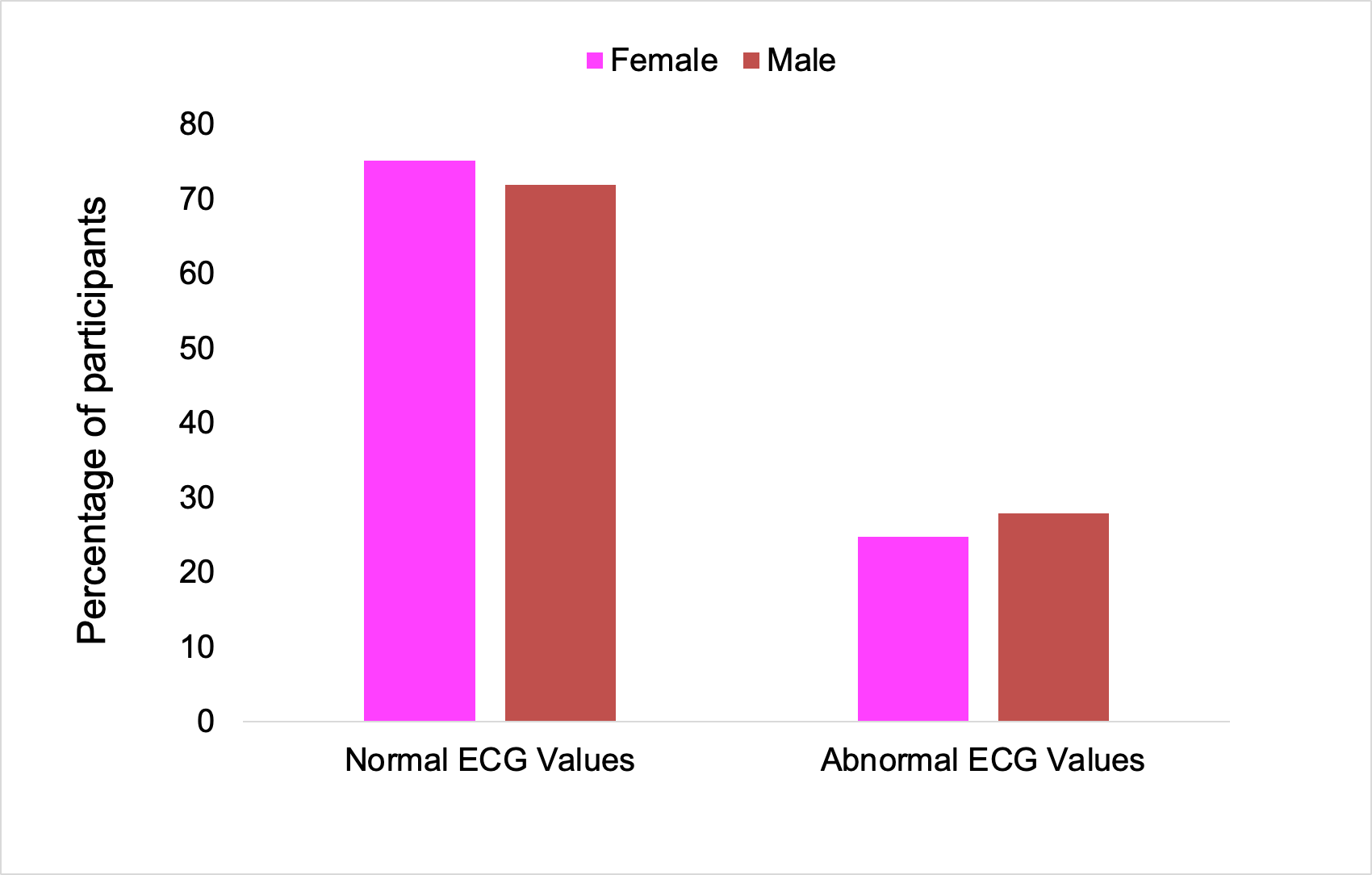


**Figure S5: Gender-based electrocardiogram (ECG) analysis**


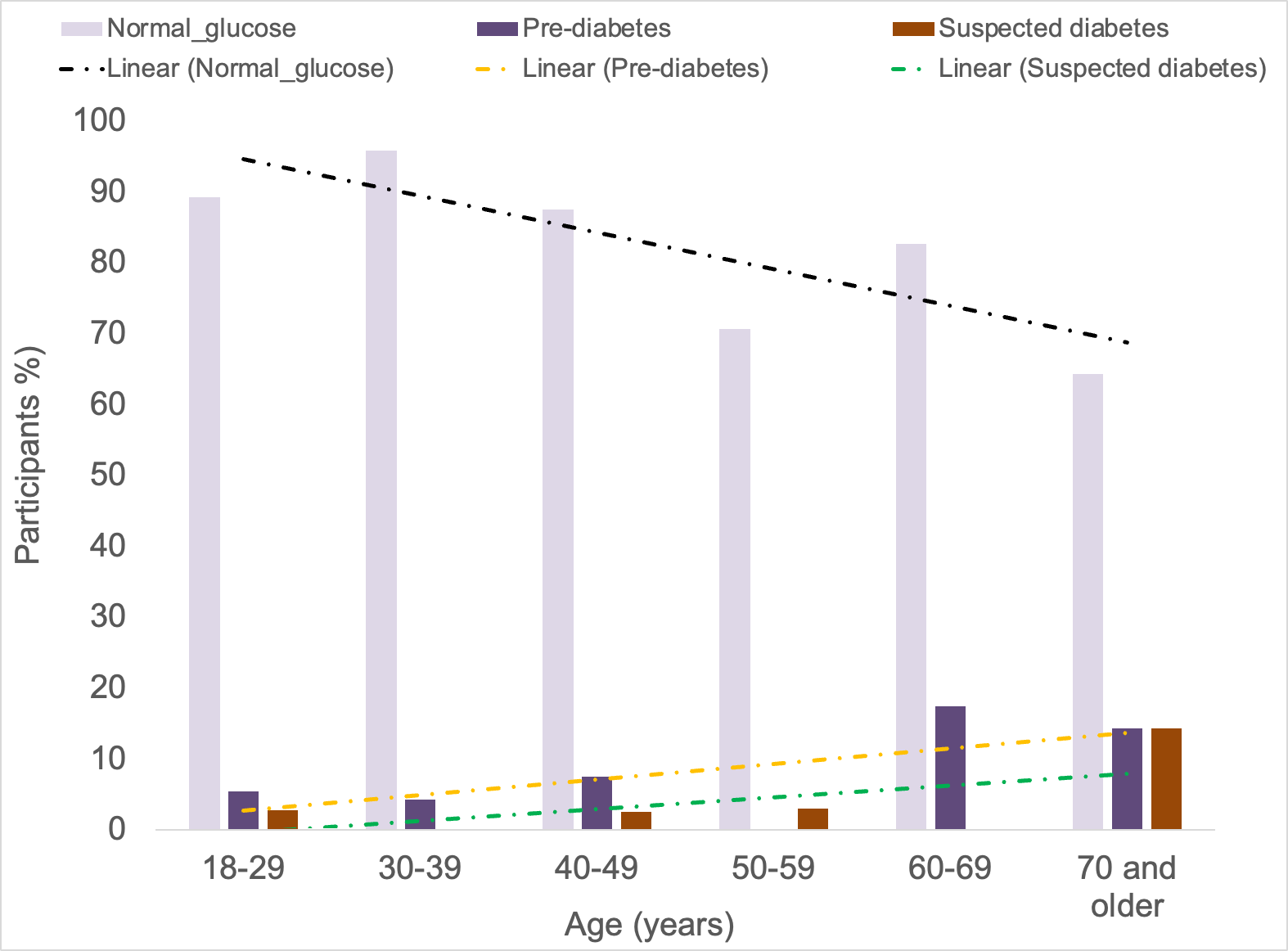


**Figure S6: Frequency of high blood glucose with age**


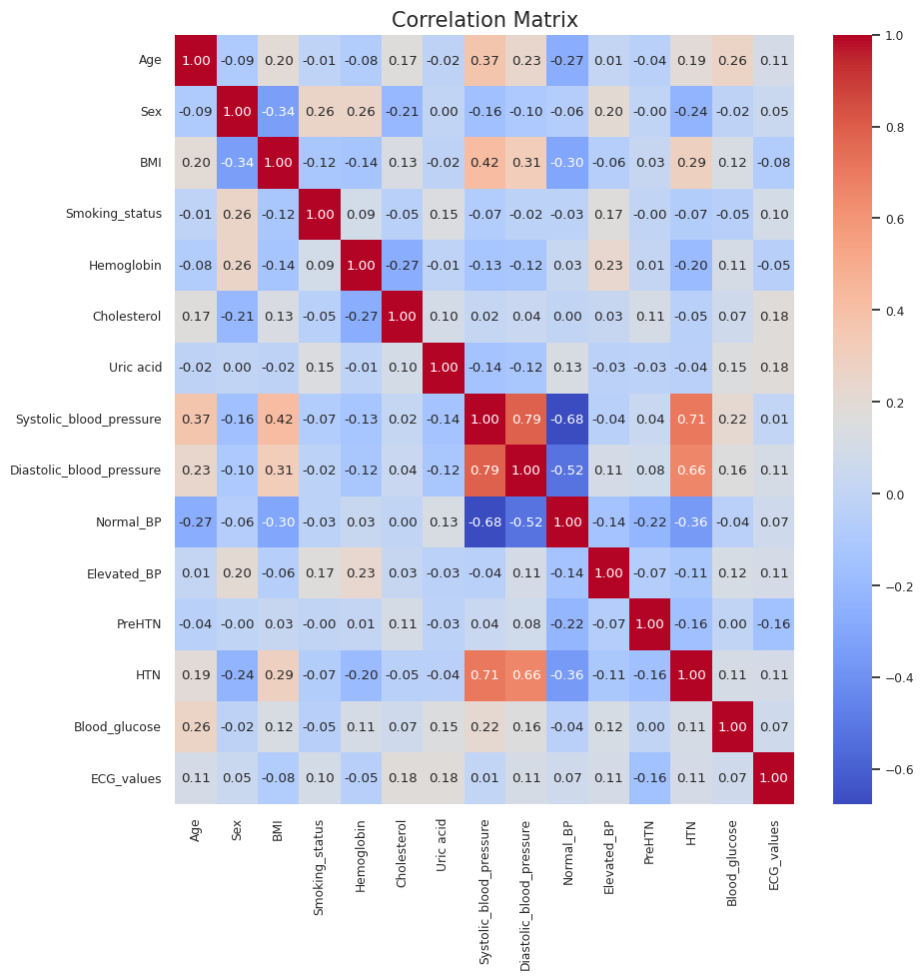


**Figure S7: Correlation matrix of variables contained in the dataset.**


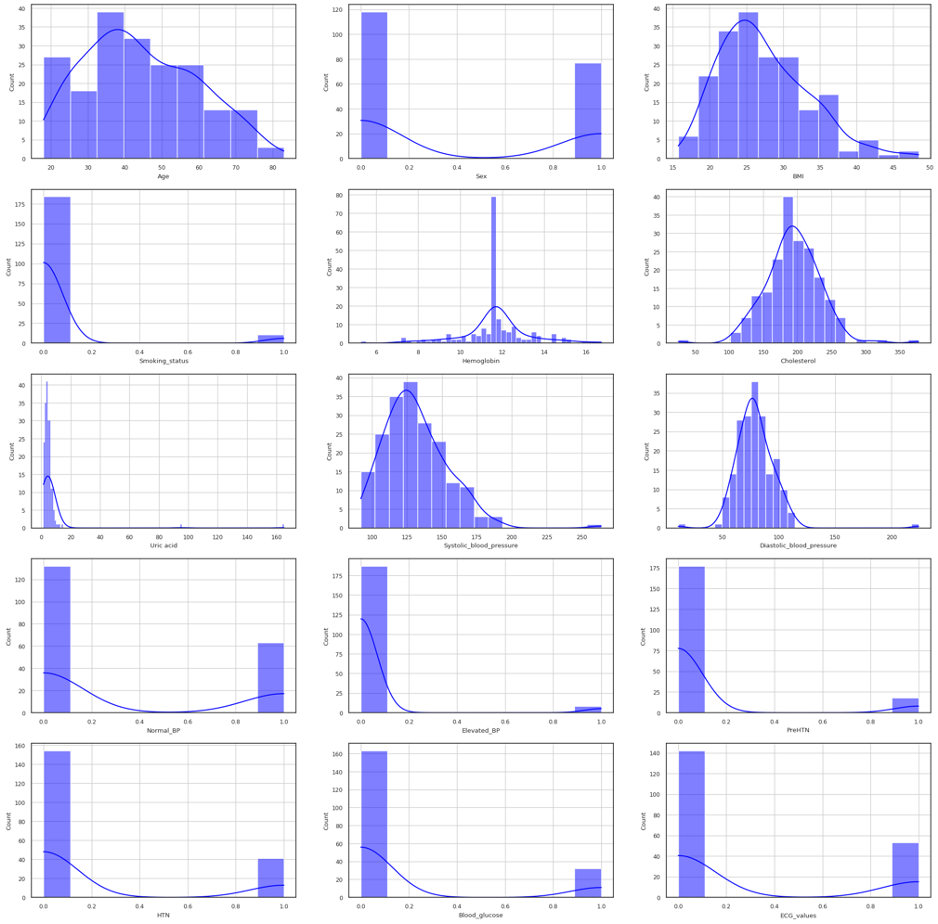


**Figure S8: Distribution of variables in cleaned dataset**


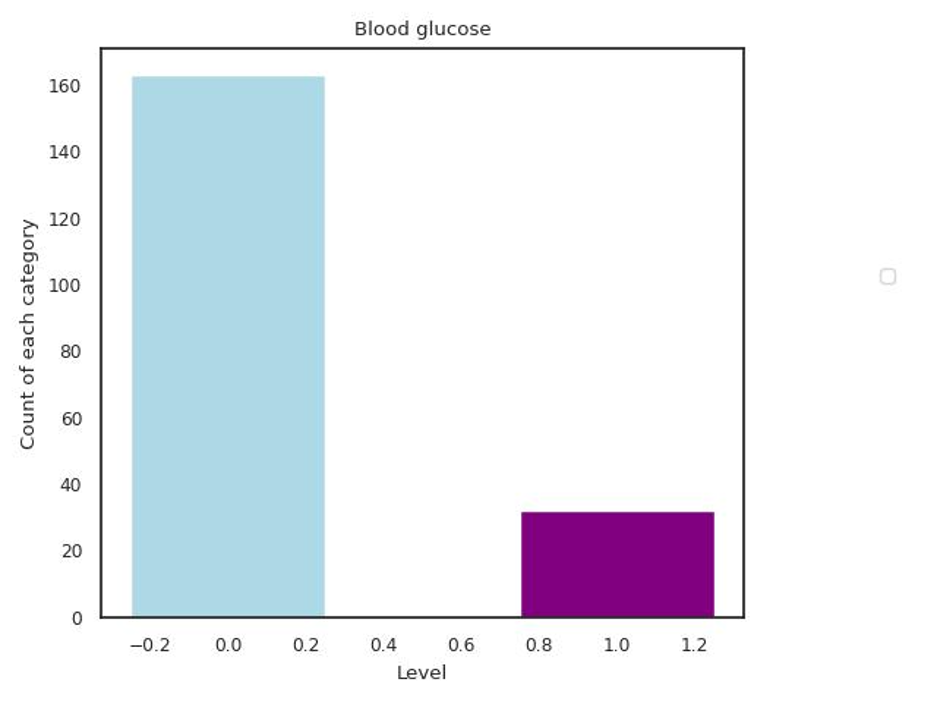


**Figure S9: Class imbalance in the outcome** (blood glucose level) variable. 163/195 (83.6%) participants had normal blood glucose {0} while 32/195 (16%) participants had high blood glucose level {1}

**Table S1: Model performance report before SMOTE rebalancing**

| **Model** | **Accuracy** | **Balanced Accuracy** | **ROC AUC** | **F1 Score** | **Time Taken (s)** |
| --- | --- | --- | --- | --- | --- |
| Random forest classifier | 0.82 | 0.50 | 0.50 | 0.74 | 0.68 |
| Dummy classifier | 0.82 | 0.50 | 0.50 | 0.74 | 0.11 |
| Ada boost classifier | 0.82 | 0.56 | 0.56 | 0.78 | 0.43 |
| SVC | 0.82 | 0.50 | 0.50 | 0.74 | 0.05 |
| Bernoulli NB | 0.82 | 0.61 | 0.61 | 0.80 | 0.04 |
| Calibrated classifier CV | 0.79 | 0.48 | 0.48 | 0.73 | 0.22 |
| K neighbors classifier | 0.79 | 0.48 | 0.48 | 0.73 | 0.07 |
| Bagging classifier | 0.79 | 0.48 | 0.48 | 0.73 | 0.20 |
| Passive aggressive classifier | 0.79 | 0.65 | 0.65 | 0.79 | 0.04 |
| Quadratic discriminant analysis | 0.77 | 0.52 | 0.52 | 0.74 | 0.06 |
| Extra trees classifier | 0.77 | 0.47 | 0.47 | 0.71 | 1.01 |
| Ridge classifier | 0.77 | 0.47 | 0.47 | 0.71 | 0.03 |
| Ridge classifier CV | 0.77 | 0.47 | 0.47 | 0.71 | 0.03 |
| XGB classifier | 0.77 | 0.47 | 0.47 | 0.71 | 0.40 |
| LGBM classifier | 0.77 | 0.47 | 0.47 | 0.71 | 0.13 |
| Linear discriminant Analysis | 0.74 | 0.51 | 0.51 | 0.73 | 0.10 |
| Gaussian NB | 0.74 | 0.51 | 0.51 | 0.73 | 0.06 |
| SGD classifier | 0.74 | 0.56 | 0.56 | 0.74 | 0.05 |
| Linear SVC | 0.74 | 0.51 | 0.51 | 0.73 | 0.07 |
| Perceptron | 0.72 | 0.55 | 0.55 | 0.73 | 0.08 |
| Logistic regression | 0.72 | 0.44 | 0.44 | 0.69 | 0.08 |
| Decision tree classifier | 0.69 | 0.53 | 0.53 | 0.71 | 0.06 |
| Label spreading | 0.62 | 0.43 | 0.43 | 0.64 | 0.10 |
| Nearest centroid | 0.62 | 0.60 | 0.60 | 0.66 | 0.09 |
| Label propagation | 0.59 | 0.42 | 0.42 | 0.62 | 0.07 |

ROC-AUC: receiver operating characteristic–area under the curve.

SVC: support vector classification

LGBM: light gradient boosting machine

XGB: extreme gradient boosting

^e^CV: cross validation

NB: Naive Bayes

SGD: stochastic gradient descent

**Table S2: Performance of model classifiers following the synthetic minority oversampling technique rebalancing.**

| **Model** | **Accuracy** | **Balanced accuracy** | **ROC-AUC^a^** | **F_1_-score** | **Time taken (s)** |
| --- | --- | --- | --- | --- | --- |
| Random forest classifier | 0.92 | 0.92 | 0.92 | 0.92 | 0.23 |
| NuSVC | 0.91 | 0.90 | 0.90 | 0.91 | 0.02 |
| LGBM classifier | 0.89 | 0.89 | 0.89 | 0.89 | 0.11 |
| Extra trees classifier | 0.88 | 0.87 | 0.87 | 0.88 | 0.16 |
| Label propagation | 0.86 | 0.86 | 0.86 | 0.86 | 0.03 |
| Label spreading | 0.86 | 0.86 | 0.86 | 0.86 | 0.04 |
| XGB classifier | 0.82 | 0.82 | 0.82 | 0.82 | 0.09 |
| K neighbors classifier | 0.82 | 0.81 | 0.81 | 0.81 | 0.02 |
| Bagging classifier | 0.77 | 0.77 | 0.77 | 0.77 | 0.06 |
| SVC | 0.76 | 0.76 | 0.76 | 0.76 | 0.03 |
| Decision tree classifier | 0.74 | 0.74 | 0.74 | 0.74 | 0.02 |
| Ada boost classifier | 0.73 | 0.73 | 0.73 | 0.73 | 0.16 |
| Ridge classifier CV | 0.71 | 0.71 | 0.71 | 0.71 | 0.02 |
| Ridge classifier | 0.71 | 0.71 | 0.71 | 0.71 | 0.02 |
| Linear discriminant analysis | 0.70 | 0.70 | 0.70 | 0.70 | 0.03 |
| Quadratic discriminant analysis | 0.68 | 0.69 | 0.69 | 0.68 | 0.03 |
| Calibrated classifier CV | 0.68 | 0.68 | 0.68 | 0.68 | 0.06 |
| Passive aggressive classifier | 0.68 | 0.66 | 0.66 | 0.65 | 0.03 |
| Linear SVC | 0.67 | 0.67 | 0.67 | 0.67 | 0.05 |
| Logistic regression | 0.67 | 0.67 | 0.67 | 0.67 | 0.02 |
| Bernoulli NB | 0.65 | 0.65 | 0.65 | 0.65 | 0.02 |
| SGD classifier | 0.62 | 0.62 | 0.62 | 0.62 | 0.02 |
| Perceptron | 0.62 | 0.61 | 0.61 | 0.61 | 0.02 |
| Gaussian NB | 0.58 | 0.59 | 0.59 | 0.56 | 0.03 |
| Nearest centroid | 0.56 | 0.56 | 0.56 | 0.56 | 0.02 |
| Dummy classifier | 0.47 | 0.50 | 0.50 | 0.30 | 0.02 |

ROC-AUC: receiver operating characteristic–area under the curve.

SVC: support vector classification

LGBM: light gradient boosting machine

XGB: extreme gradient boosting

^e^CV: cross validation

NB: Naive Bayes

SGD: stochastic gradient descent

**Hyperparameter optimization before SMOTE rebalancing**

precision recall f1-score support

0 0.82 1.00 0.90 32

1 0.00 0.00 0.00 7

accuracy 0.82 39

macro avg 0.41 0.50 0.45 39

weighted avg 0.67 0.82 0.74 39

Best Params: {'max_depth': 30, 'min_samples_leaf': 1, 'min_samples_split': 2}

Best F1 Score: 0.15

Test F1 Score: 0.8205128205128205

**Hyperparameter optimization after SMOTE rebalancing**

precision recall f1-score support

0 0.87 0.87 0.87 31

1 0.89 0.89 0.89 35

accuracy 0.88 66

macro avg 0.88 0.88 0.88 66

weighted avg 0.88 0.88 0.88 66

Best Params: {'max_depth': 10, 'min_samples_leaf': 1, 'min_samples_split': 2}

Best F1 Score: 0.8730174729485075

Test F1 Score: 0.8787878787878788
